# Activity and case-mix changes in a medical ICU after the geographical transfer of a third-level university hospital

**DOI:** 10.1186/cc12443

**Published:** 2013-03-19

**Authors:** JC Cebrián, FM Monsalve, JB Bonastre

**Affiliations:** 1 Hospital Universitario y Politecnico La Fe, Valencia, Spain

## Introduction

Information about big hospital geographical transfer is scarce in the medical literature. On 20 February 2011 our hospital (in fact, a big university complex) was transferred from their previous location in the north-center of our city towards a new southern peripheral, geographical location. This transfer has been done without any changes in assisted population or nursing/medical staff. The only change was a slight increase in bed number (21 to 24). Our aim is to analyze changes in activity indexes (length of stay, occupancy rate, and so forth) and case mix (origin, previous quality of life and NYHA score, main diagnostic groups, severity scores, in-ICU and in-hospital mortality).

## Methods

To compare our number of admissions, related activity and case-mix indicators 1 year before and after the geographical change was done. We analyzed our whole number of patients admitted to the ICU. We used the chi-square test for categorical variables and one-way analysis of variance for quantitative data. Minitab and Statbas statistical programs were used. We plotted activity data using the Barber-Johnson 1 diagram.

## Results

A total of 2,774 cases (63% males; mean age 61 years) were admitted to our ICU during the period (1 year before and after the transfer). No differences between both groups were founded in demographic data, Knaus score and NYHA status. Regarding their origin, we found more patients admitted from other hospital centers (20 vs. 29%; *P *0.001). APACHE II score increased from 17.24 to 19.08% (*P *0.001) and a slight increase change in SAPS 3 score was also found (52.29 to 53.75; *P *0.01). Our in-ICU mortality remains lower (15.5 to 15.6%) whereas observed mortality decreased (22.37 to 19.88%; *P *0.001). An increase in our neurologic patients has been the most consistent change regarding diagnostic groups. The activity indexes show a slightly decrease in occupancy rate (79.2 vs. 76.8).

## Conclusion

According to the previous data our ICU seems to perform better in the new location with a decrease in the standardized mortality rate. On the other hand, we are admitting more patients transferred from other hospitals. A better occupancy rate was found.
